# Food system actor perspectives on future-proofing European food systems through plant breeding

**DOI:** 10.1038/s41598-023-32207-1

**Published:** 2023-04-03

**Authors:** S. Stetkiewicz, J. Menary, A. Nair, M. Rufino, A. R. H. Fischer, M. Cornelissen, A. Guichaoua, P. Jorasch, S. Lemarié, A. K. Nanda, R. Wilhelm, J. A. C. Davies

**Affiliations:** 1 Lancaster University, Lancaster, LA1 4YX Lancashire UK; 2 Marketing and Consumer Behaviour Group, Wageningen University, 6700 EW Wageningen, The Netherlands; 3 BASF Innovation Center Gent, 9052 Gent, Belgium; 4 ACTA -The Agricultural Technical Institutes, 75595 Paris, France; 5 Euroseeds, 1000 Brussels, Belgium; 6 Université Grenoble Alpes, CNRS, INRA, Grenoble INP, 38400 Saint-Martin-d’Hères, France; 7 ‘Plants for the Future’ European Technology Platform, 1000 Brussels, Belgium; 8 Federal Research Centre for Cultivated Plants, Julius Kühn-Institut, 06484 Quedlinburg, Germany

**Keywords:** Plant breeding, Climate-change adaptation, Sustainability

## Abstract

Crop improvement is a key innovation area in the pursuit of sustainable food systems. However, realising its potential requires integration of the needs and priorities of all agri-food chain stakeholders. In this study, we provide a multi-stakeholder perspective on the role of crop improvement in future-proofing the European food system. We engaged agri-business, farm- and consumer-level stakeholders, and plant scientists through an online survey and focus groups. Four of each group’s top five priorities were shared and related to environmental sustainability goals (water, nitrogen and phosphorus efficiency, and heat stress). Consensus was identified on issues including considering existing alternatives to plant breeding (e.g. management strategies), minimising trade-offs, and addressing geographical variation in needs. We conducted a rapid evidence synthesis on the impacts of priority crop improvement options, highlighting the urgent need for further research examining downstream sustainability impacts to identify concrete targets for plant breeding innovation as a food systems solution.

## Introduction

Future-proofing agriculture is a major global priority^1,2^ given the agronomic challenges under a changing climate and declining natural resources, the rising needs of a growing global population with changing diets, new targets for a growing bioeconomy, and the necessity to reduce agriculture-driven environmental degradation. Plant breeding offers one important area of focus for future-proofing food systems^3^. In recent decades, in line with growing global prioritisation of sustainability, plant breeding has made advances in increasing crop resilience to abiotic stresses such as heat^4^, drought^5^ and soil salinity^6,7^ and improving nutrient^8,9^ and water use efficiency^10^, contributing to a history of breeding increasing crop yields^11^. These improvements at the plant level offer the potential to help agriculture remain productive in the face of climate change, water scarcity, and adverse growing conditions whilst reducing fertiliser use and other inputs.

Despite clear evidence of tangible benefits from crop improvement, such as yield gains^12^, we lack an integrated multi-stakeholder food system view on the potential for plant breeding to contribute towards a resilient and healthy food system aligned with sustainability goals. Gaining such a systemic understanding of in-plant innovations and their associated benefits, pitfalls, and unintended consequences is vital to guide research, development, and policies that contribute to future-proofing agriculture; the more so given the complexity of food systems, the diverse array of stakeholders engaged in them, and their multiple drivers and outcomes. Whilst participatory plant breeding approaches have been deployed in some contexts^13^, the majority of breeding efforts do not take a holistic approach to incorporating the views and knowledge of wider food system actors and outcomes. Understanding whether food system stakeholder views, needs and priorities on plant breeding are aligned or in tension is essential in directing innovation and key to its subsequent success and sustainability. Embedding stakeholder needs in innovation development is a crucial step towards mitigating the types of power dynamics and sometimes perverse outcomes which have been critiqued from the Green Revolution^14–16^ and avoiding issues such as ‘pesticide-lock in’, which have been reported in relation to slow uptake of low-input varieties in the past^17^.

**Figure 1:**
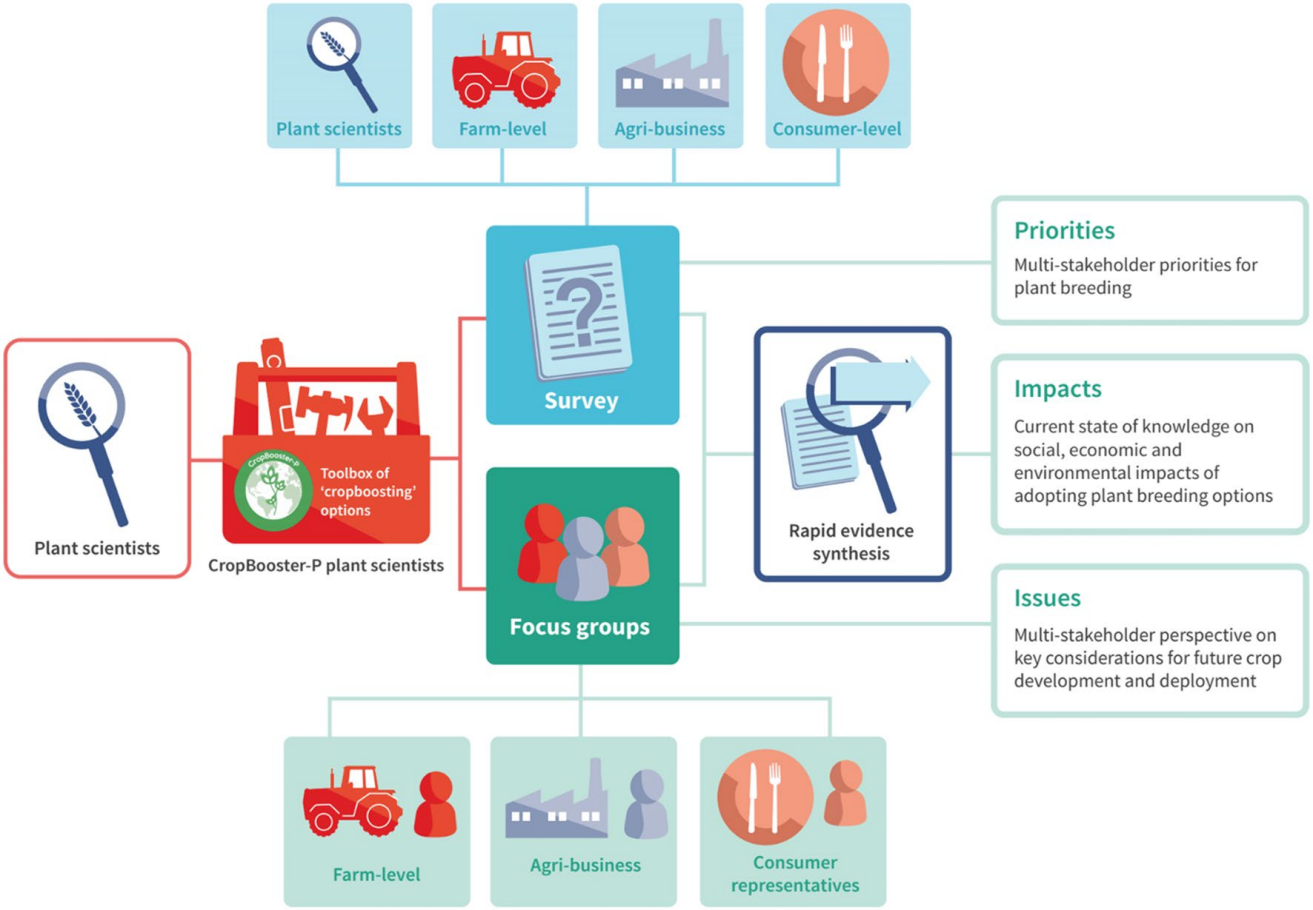
Project approach. Plant science insights (left) fed into stakeholder survey (top) and focus groups (bottom). Outputs were combined with rapid evidence synthesis (right) to provide priorities, impacts, and issues around plant breeding.

This paper provides a first food system-based multi-stakeholder perspective on the priorities of plant breeding for future-proofing crop production in Europe, the key broader, systemic issues that need to be considered, and the potential social, economic and environmental impacts of in-crop improvements. To achieve this, we combine evidence from a mixed-method three stranded approach, as shown in Fig. 1, engaging four key groups of stakeholders: farm-level (farmers, farmer representatives, NGOs and policy makers working on agri-environmental issues), agri-business (including plant breeders and seed companies), consumer-level (consumers and consumer experts), and plant scientists. We triangulate the evidence by combining survey data to establish priorities in a larger group of stakeholders, rapid evidence reviews to represent the scientific state-of-the-art on impacts of the crop breeding options with more elaborated in-depth insights from expert focus groups on societal issues.

## Results

### Priorities

**Figure 2:**
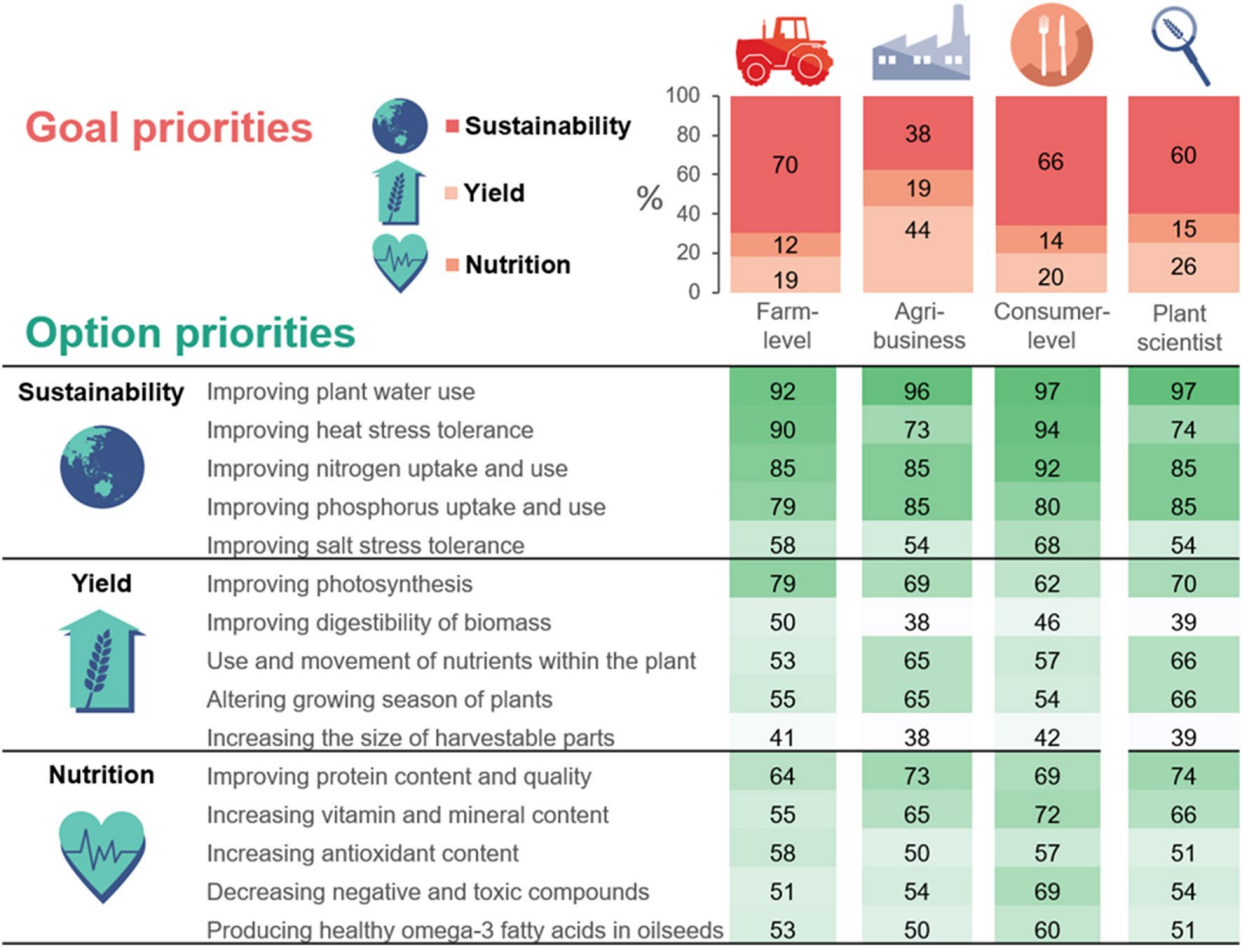
Survey respondent priorities for options and goals. The percentage of respondents from each stakeholder group selecting a given goal as their top priority is indicated in red (top right). The percentage of respondents from each stakeholder group selecting a given CropBooster option as ‘important’ or ‘very important’ is indicated in green, with darker green shading indicating a higher proportion of respondents expressing a preference for a given option.

The 254 respondents who completed the survey were presented with environmental sustainability, yield, or nutrition as potential overarching plant breeding goals. The majority of farm-level (70%), consumer-level (66%), and plant scientist (60%) respondents to the survey selected sustainability as most important for crop improvement in Europe. Agri-business respondents were more evenly split between yield (44%) and sustainability (38%) as most important (Fig. 2). Nutrition was the least frequently selected priority for every stakeholder group, with less than 20% selecting it as most important in any group; however, when nutrition-related crop breeding options were presented individually, over half of participants selected these as important or very important. This importance is reflected in the relatively high percentage of respondents choosing nutrition as the second most important goal: 58% of farm-level, 44% of agri-business, 49% of plant scientist, and 46% of consumer-level respondents. Respondents from 15 European countries completed the survey, with a majority coming from the UK (83), followed by Italy (31) and France (15)—for more information on the survey responses, including a demographic break-down, see Supplementary 2.

When examining more specific options for plant breeding—based on areas identified by plant breeding researchers as having significant potential value for future-proofing European crop production (with a focus on abiotic stress)—the 201 stakeholders across the food system completing this survey section broadly agree that crop improvements which enhance environmental sustainability are important for future-proofing the food system in Europe. Four of the five options most commonly identified as important are shared across stakeholder groups and fall within the category of environmental sustainability, namely: ‘improving plant water use,’ ‘improving heat stress tolerance,’ ‘improving Nitrogen uptake and use,’ and ‘improving phosphorus uptake and use’ (Fig. 2). These options were also relevant to several important issues in stakeholder focus groups where a key overarching theme around the need for resilience to climate change was identified (See Fig. 4). The fifth option most frequently selected as important varied between stakeholder groups, with priority given to ‘improving photosynthesis’ by the farm-level stakeholders, ‘improving protein content and quality’ by agri-business and plant scientist groups, and ‘increasing vitamin and mineral content’ by those in the consumer-level group.

Very few options where plant breeding could help improve environmental sustainability, yield or nutrition were considered a low priority for future-proofing the European food system. The lowest priority categories were improving the digestibility of biomass and increasing the size of harvestable parts of the crop. However, a substantial minority did select these options as important or very important (43% and 40%, respectively). Focus group discussions with stakeholders around these options suggest digestibility of biomass may have been perceived as less important due to stakeholders prioritising food over biofuel. With respect to increasing the size of harvestable parts, concerns were raised regarding the impact on product quality, harvest, processing, and biomechanical stress on plant structures (e.g. overly large fruits causing the breakage of stems), though potential increases in profit were also noted (see https://doi.org/10.17026/dans-xp4-j8t7).

Improving plant water use efficiency, improving photosynthesis and increasing protein content and quality were identified as the priority crop improvements within their respective goal categories. They had the highest average percentage of respondents across the food system selecting them as important/very important with 95.5%, 70%, and 70%, respectively.

### Impacts

**Figure 3:**
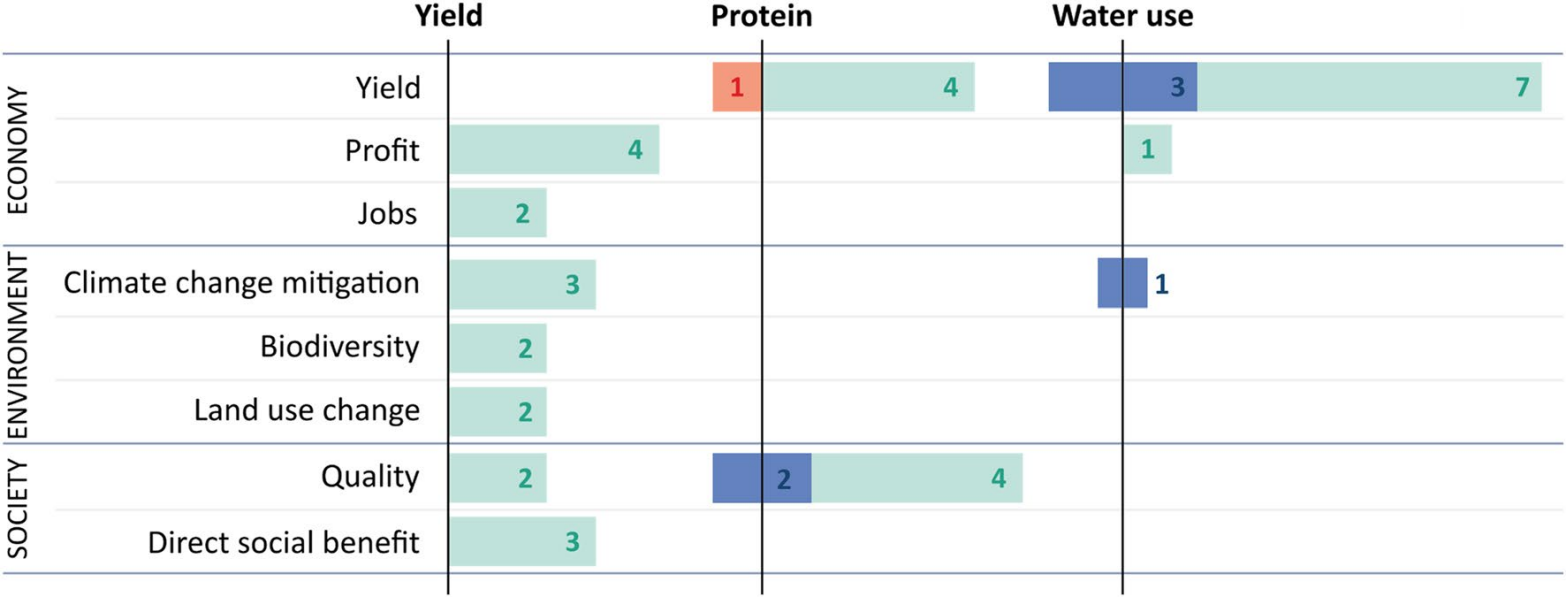
Summary of the impact areas and direction of change identified in the rapid evidence syntheses—position indicates negative (red), neutral (blue), or positive (green) scores. Numbers indicate the number of papers assessed with a given result. The following definitions were used for impact classification: Benefit (green): Positive changes in the impact being assessed were reported in the literature; Neutral or Variable (blue): No clear changes in the impact being assessed were reported in the literature, or some combination of beneficial, neutral, and/or disbenefit impacts were reported in the literature, with no clear general direction; Disbenefit (red): Negative changes in the impact being assessed were reported in the literature. ‘Direct social benefit’ (also referred to in the literature as ‘social benefit’ or ‘social welfare’ the total value to society of the production of a particular good—in this case, plant breeding.

The rapid evidence synthesis aimed to understand the potential environmental sustainability impacts of the three crop improvement options assessed based on their importance to surveyed stakeholders: water use, protein content/quality, and yield increase (which replaced photosynthesis due to a lack of literature on the latter which reported on downstream impacts). However, few studies were found which have attempted to analyse or quantify the economic, social, and environmental impacts of these options. A total of 21 papers were reviewed in depth following initial screening of 1,398 papers (10 papers were retained relating to water use; 5 for yield increase; and 6 for protein content and quality—see Supplementary 3) most of which were removed as downstream impacts were not explicitly reported on. Nearly half (9 out of 21) of the papers reviewed reported on a single impact indicator only (see Fig. 3). The majority of papers relating to water use focused only on yield impacts (8 out of 10 papers). Protein-related papers frequently reported on two indicators, both quality and yields (5 out of 6 papers). Four of the five papers reviewed relating to yield reported on three or more indicators. Across all 21 papers, quality was the second-most frequently assessed impact, with a total of 8 papers reporting on this indicator (two in the yield and six in the protein categories). The prevalence of yield-related papers in the water use and protein categories, along with the small number of papers retained in the yield review (and the fact two of these are ‘grey literature’ funded by plant breeding associations), points to the ongoing importance of yield as a plant breeding aim, despite the fact that the downstream impacts of improving yield were not well-elaborated in the reviewed literature.

Only one study reported overall disbenefits of breeding; a paper assessing potential yield loss due to climate change^18^ (see Supplementary 3, protein section). No studies reviewed focused specifically on assessing potential disbenefits of in-plant solutions, such as trade-offs between reducing nutritional quality and yield, or reduced food sovereignty with increased reliance on upstream industry.

More studies are needed that systematically quantify the benefits and trade-offs of plant breeding solutions to form a fuller business case and guide the development and deployment of improved crops. In particular, while focus group participants stressed the need to consider resilience to climate change, few studies reported on the broader environmental impacts of these breeding goals. Stakeholder concerns regarding market driven needs and value chain impacts were echoed in only the minority of studies that looked at economic indicators. Yield stability over the long term, a trait highlighted by both farm-level and agri-business stakeholders, was also not widely assessed, with the maximum length of the field trials included in the literature syntheses being 4 years^19^.

### Emerging issues

**Figure 4:**
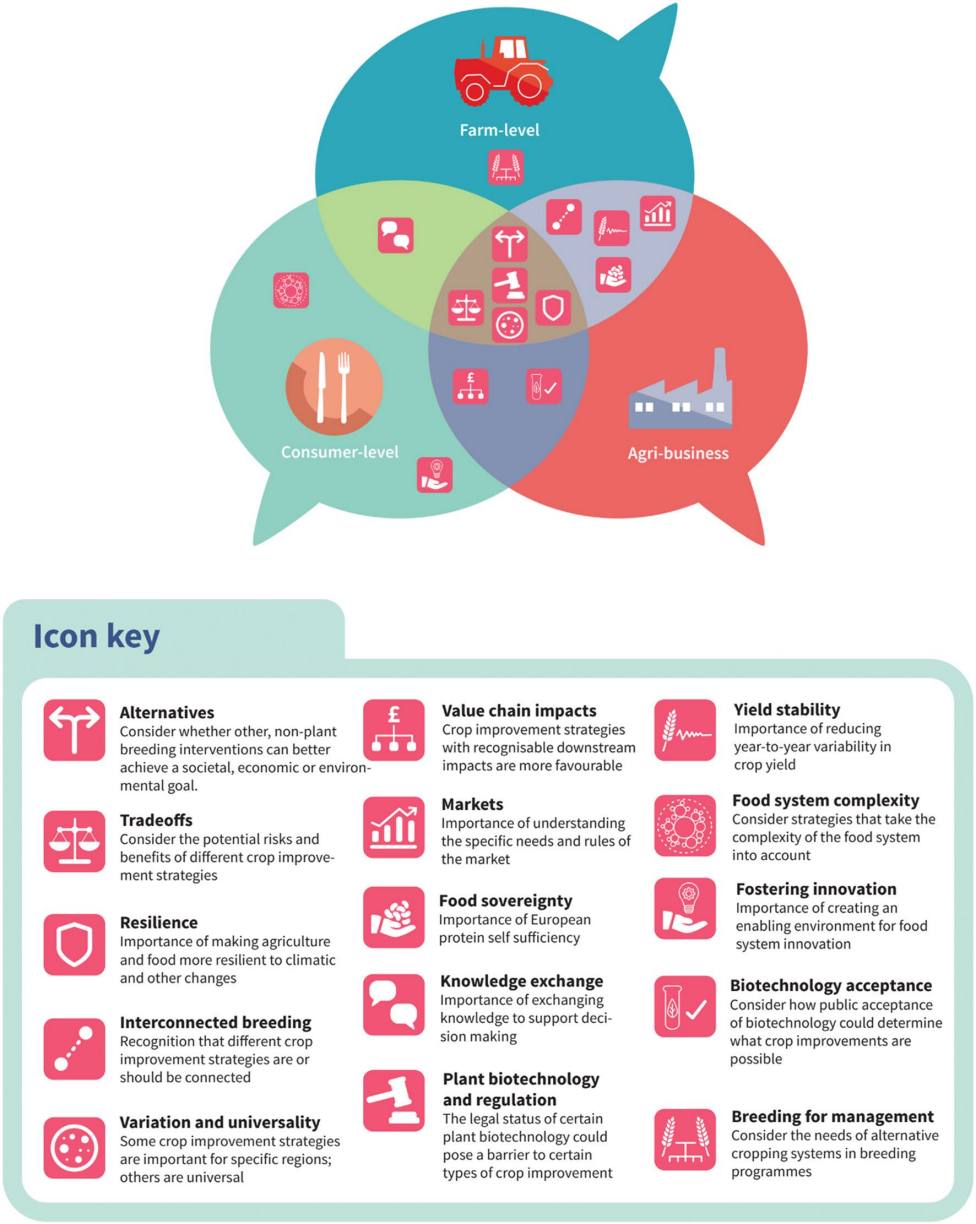
Key themes arising from focus group discussions. Themes icons are used inside the speech bubbles to indicate which stakeholder group(s) raised each of the key themes listed in the icon key.

Stakeholder focus groups were conducted to provide in-depth, qualitative data to complement and provide context to the quantitative data described above, and gather insights into issues of importance to consider in developing breeding programmes. Five key issues were identified and shared across the agri-business, farm- and consumer-level stakeholders in online focus groups, as shown in Fig. 4 and described as follows.

1. Alternatives to plant breeding options. Assessing existing alternatives to plant breeding was considered important across all groups. The experts stressed that it is important that alternative means of reaching the same outcomes (i.e. changing farm management practice, use of heritage crops, and changing diets) are explored and weighed against and in complement to plant breeding solutions (see Supplementary 4).
2. Minimising trade-offs in plant breeding. Minimising trade-offs when breeding was a significant concern, for example, breeding to reduce negative and toxic compounds potentially being traded-off against crop pest and disease resistance, thus requiring more pesticide use.
3. Variation and universality in plant breeding needs: Many stakeholders raised the importance of understanding variation and universality in plant breeding needs, highlighting that specific issues, such as salt stress vary in importance geographically and/or temporally. In contrast, others, such as heat stress, will affect a wide range of crops and geographical regions.
4. Resilience: The need to build resilience into food systems was stressed, particularly concerning climate change and the extreme weather events expected to increase in the coming decades.
5. Plant biotechnology and regulation: The overarching regulatory framework within which plant breeding and plant biotechnology operate was also raised by stakeholders who wondered what plant breeding gains were realistically achievable within the current EU breeding restrictions and what the future might bring for plant biotechnology.

There were many other issues identified by only one or two groups, which have importance for the future success of crop breeding (Fig. 2). For example, the farm-level group discussed the need to breed crops to align with sustainable farm management. They discussed ideas such as breeding pairs of crops together specifically to be sown in an inter-cropping system, which could increase the efficiency and attractiveness of such systems and bring the related benefits of reduced input needs. Those in the consumer-level group discussed the need for regulation that fostered innovation while considering associated risks. They mentioned that regulation needs to be updated and made more proactive in order not to hinder innovation in plant breeding. Both farm- and consumer-level groups highlighted the need for improved communication and knowledge exchange, with better integration between scientists, policymakers, farmers, and consumers, as key to improving sustainable scientific advances, policy outcomes, and informed-decision making. Several issues were shared between farm-level and agri-business stakeholders, including the importance of striving for interconnected breeding plans. For example, breeding new crops that meet multiple needs to cope with future climate uncertainties (e.g. crops with improved water use, improved heat stress tolerance, and improved Nitrogen use). Consumer-level and agri-business stakeholders also shared concerns about consumer (non) acceptance of plant biotechnology and the need to engage with consumers around this issue. When asked what additional items should be focused on in plant breeding beyond those options presented in the project, both focus group and survey participants raised the need to consider future pest and disease pressure as key breeding issues (see Supplementary 2 and 4).

## Discussion

This study highlights several overarching agreements between diverse food-system stakeholders regarding what is necessary to future-proof crops for the European food system, which could inform the global approach to plant breeding for sustainability. Stakeholders broadly agree about the importance of environmental sustainability-related crop breeding options, with a particular consensus around the need to improve plant water use to build resilience in preparation for more extreme climatic conditions. Shared concerns regarding variation in the utility of the options presented, existing alternatives to plant breeding solutions, and the need to avoid trade-offs must be incorporated into plant breeding programmes’ prioritisation and strategic planning.

Whilst many priorities between stakeholders are aligned, this multi-stakeholder perspective study highlights that a negotiated agenda for plant breeding is needed: one which brings together stakeholders from across the food system to strategically prioritise crop breeding objectives and consider their role within a wider suite of actions. A holistic approach to plant breeding is needed which takes into account several interlinked breeding goals, and assesses potential trade-offs, synergies, and alternatives across a wide range of transparent sustainability metrics, with aligned incentives, to encourage sustainable and effective breeding innovations. This type of systems-based breeding approach^20^ can, by providing context and input from multiple actors, produce effective outputs which achieve a variety of sustainability goals in concert. Investment in plant breeding is substantial; in 2016–2017, to take examples of European relevance, German seed company KWS spent €190 million on R&D, while French seed company Vilmorin invested €240 million in research; corresponding to R&D intensities of 14 and 15%, respectively^21^, and there is evidence this is increasing further, with KWS reporting spending €286 million on R&D in 2021-22, an R&D intensity of 18.6%^22^. It is therefore particularly important to ensure that such efforts are targeted to the needs of the food system and contextualised against the cost, efficacy and impacts of alternative methods.

The rapid evidence synthesis conducted stresses the need for further research that examines the wider impacts of in-plant solutions beyond yields, compares and contextualises these to other alternative solutions, and is open to examining potential disbenefits to the food system. Very few studies have attempted to directly quantify or detail the effects that adopting in-crop solutions have for the food system. For example, to what extent can crop improvements help reduce on-farm greenhouse gas emissions or help sequester carbon in the soil? To what extent can it help protect water resources by reducing irrigation and reducing fertilizer run-off? To what extent can crop improvement help reduce micro-nutritional deficits in socioeconomically deprived groups of society?

Bringing together these three data sources (focus groups, surveys, and rapid evidence synthesis) for a range of stakeholders involved across the food system highlights both broad agreements on the need to prioritise sustainability in plant breeding, as well as context and group-specific issues of importance, such as regional and crop-level variation in need and the potential to breed for specific farm management contexts. Examples raised include the difference in drought concern varying geographically within Europe, or the potential to breed specifically for use in intercropping systems (for more detail, see Supplementary 4). These differences in aspects raised across stakeholder groups underline the need to include various voices in prioritisation and planning exercises for plant breeding. While this study provides a first, systemic insight from key groups across the food system, further work is needed to bring additional stakeholder groups of relevance into the conversation, including those involved in the processing, storage, and retail sectors. Broadening the dialogue between plant breeders and other stakeholders is crucial for providing a ground-truthed direction for future-proofing our crops for the food system.

## Methods

### Design

The study used a sequential mixed-methods design with three stages: a survey, rapid evidence synthesis, and stakeholder focus groups (Fig. 1). Ethical approval was granted by Lancaster University Faculty of Science and Technology Research Ethics Committee (FST19070), and all methods were performed in accordance with the relevant guidelines and regulations. Informed consent was obtained from all participants prior to data collection.

Participants for the survey and focus groups were purposively sampled from three pre-defined stakeholder groups:

- Farm-level
  o Farmers, farmer associations or cooperative representatives
  o Farm- or agri-environment-focused non-governmental organisation representatives
  o Farm- or agriculture-focused policy makers
- Agri-businesses
  o Plant breeding representatives
  o Food producer/processor association representatives
  o Other agri-business stakeholders (survey only)
- Consumer-level
  o Consumer group representatives
  o Consumer experts
  o Consumers (survey only)

To present stakeholders with the discussion topics, we developed crop improvement “option cards”, which displayed 15 potential crop improvement options organised into three categories: yield, nutrition and sustainability. These were used in both the survey and focus groups to appraise different crop improvement strategies in quantitative and qualitative terms. One side offered a simple explanation of the improvement (e.g. improving plant water use) and the other an example of that improvement being applied through research (see [Supplementary 5]). A blank card labelled “Option Card #16” was provided to allow for opinions missing from other cards.

A brief description of the survey and focus group methods follows, however detailed reporting on both can be found in Supplementary 1.

### Priorities survey

#### Participants

Participants of the survey were volunteer stakeholders identified through the professional networks of the CropBooster-P consortium and through snowball sampling. The Cropbooster-P consortium consisted of a variety of stakeholders based in the EU and the UK including academics and representatives of farmer, plant breeder and seed producer groups. Participants self-identified as belonging to each one of the predefined groups. A total of 324 participants took part in the online survey (288 in English, 22 in French, and 14 in German). For more information, see Supplementary 1 and 3.

#### Survey instrument

The survey was designed to identify which of the CropBooster-P crop improvement options were prioritised among a wide group of European food system stakeholders. The survey consisted primarily of closed questions, with some open-ended qualitative questions included to elicit more complex responses to key questions. It was programmed and administered using *Qualtrics* (www.qualtrics.com). Reporting follows CHERRIES guidelines^23^ (See Supplementary 6 for a copy of the survey in English). To access as many participants as feasible, it was translated and piloted in German and French using a modified TRAPD method^24^.

Preferences for crop improvement goals (e.g. sustainability) were elicited on a 1–3 scale, with 1 being the most preferred, using a forced ranking. Preferences for crop improvement options (e.g. increasing plant water use) was assessed on a single item Likert scale labelled 1:‘Very important’; 2: ‘Important’; 3: ‘Neither important nor unimportant’; 4: ‘Unimportant’; 5: ‘Very unimportant’ 6:‘Don’t know’. Rating was selected over forced ranking as this allows participants to indicate ties, and to rate as many options high or low as they prefer.

#### Analysis

For each stakeholder group, the total number of valid responses was used to analyse: (1) goal prioritisation and (2) the option prioritisation questions.

The percentage of each stakeholder group ranking a given goal (yield, nutrition, or sustainability) as one (top priority), two (medium priority) and three (lowest priority) was recorded, and the most frequently selected priority goal highlighted. Data from the free text questions was exported to *Nvivo 12* and thematically analysed to identify key issues. The 15 Likert-style items relating to the 15 option cards were treated as individual responses. Each of the choices was tallied and the percentage of participants choosing each statement calculated. Differences were reviewed for: top goal priority, and between stakeholder groups.

### Impacts: rapid evidence synthesis

A rapid evidence synthesis (RES, sometimes called rapid evidence assessments or rapid reviews) made up of three strands was used to explore the empirical impact of different crop improvement strategies. Rapid evidence syntheses provide relatively quick, tactical answers to key questions and are increasingly favoured by policymakers^25,26^. Given the range of possible combinations of crop types, location and types of impacts, the three highest-ranked options were selected from the survey priorities: (1) improving plant water use (sustainability), (2) improving photosynthesis (yield) and (3) improving protein content and quality (nutrition). Due to a lack of relevant peer-reviewed papers assessing the impact of photosynthesis on sustainability indicators, this category was broadened to focus on yield impacts more generally.

A common research question framed the evidence synthesis: “What are the social, economic and environmental impacts of improving [plant water use/yield (photosynthesis)/protein content and quality]?”. Slight adjustments were made depending on specifics of the priorities.

A query combining several multi keyword concept operationalisation was similarly created for the three synthesis categories (see Table 1). Search strings were adjusted based the specific needs of the priority in question: for example, the targeted improvement of photosynthetic pathways is only a recent field with relatively few impact studies^27^, so more generic improvement in yield—and the social, economic or environmental impacts this has—was targeted.

**Table 1:** Search string development.

	Goal	Option	Approach	Impact	Location
Example	“yield” OR “efficiency” OR “productivity”	“photosynthesis” AND “improve” OR “enhance”	“plant breeding” OR “crop improvement”	“impact” OR “benefit” AND “social” OR “economic” OR “environmental”	“Europe” OR “EU”

Overview of the search string strategy used in the Rapid Evidence Synthesis.

Identified papers were abstract and title screened on relevance and contents, and subsequently methodologically screened by the researcher leading that part of the synthesis within the project team on the basis of methodological norms in the relevant field of research. In cases of doubt experts in relevant fields were asked for advice on the quality assessment. When a given paper did not meet basic methodological criteria (e.g. because of issues with field trial design, model validation, or statistical analysis) the paper was removed and no further analysis of it undertaken. Papers were identified in the scientific literature using *Scopus* and *Web of Science*.

Initial searches yielded 1398 papers, 390 relating to water use, 491 relating to protein, and 515 relating to yield. After content and quality screening 10 papers remained for water use, 6 for protein and 3 for yield. Given the lack of peer-reviewed publications assessing impacts of yield, two additional papers in the grey literature were identified through Google-Scholar, bringing the total number of yield-relevant papers up to 5. Included papers were coded on relevant impact indicators.

### Emerging issues: stakeholder focus groups

Between April and June 2020, 10 participatory focus groups were held to identify emerging issues with the CropBooster crop improvement options, whilst also probing how plant breeding targets can be determined and what the challenges are for European agriculture.

Focus groups complement the survey and rapid evidence synthesis as they permit the generation of new ideas, the assessment of potential ideas and insights into the differences in opinion that exist between members of particular groups^28,29^. Face to face focus group protocols were adjusted to an online format to deal with coronavirus restrictions in Europe in 2020. A detailed description of these adjustments is described in Menary et al.^30^ and incorporates insights on online-specific focus group difficulties such as those reported by Tuttas^31^. Reporting follows COREQ guidelines^32^. A total of thirty five participants participated in one of ten focus groups (five with farm-level, two with agri-business, and three with consumer-level participants).

#### Key questions and prompts

A detailed semi-structured focus group protocol was developed to guide the moderator and ensure consistency and comparability between the data from each stakeholder group (for the full protocol, see [Supplementary 7]). The protocol was piloted at Lancaster University and Wageningen University (n = 16). Primary topics were:

- The biggest challenges for the European agri-food sector over the next 30 years
- The most important CropBooster option
- The least important CropBooster option
- The social, environmental or economic impacts of a particular option
- The relevance of the options for the challenges facing the European agri-food sector
- What other things should be included in the CropBooster options?

Topics were discussed around the 15 option cards also used in the survey. Participants were asked to fill in a blank option card (#16) with a crop improvement they thought was missing from the 15 option cards and this additional input was discussed at the end of the focus groups.

Participants were encouraged to discuss the relative merits of their suggestions and agree on the most important. Prompts were used to probe participant choices—or why certain options had not been mentioned.

#### Focus group analysis

Adopting a *Framework Analysis* approach^33,34^ an initial coding framework was developed by open coding of transcripts associated with each stakeholder group by the moderator responsible for that group. After these were agreed through consultation with at least one other member of the research team, the transcripts were fully coded and analysed using *NVivo* software. Emergent themes were cross-referenced by the moderators of the focus groups (AN, JM and SS) and an overview of themes was discussed within the wider research team. Mutual language was agreed upon for the purposes of illustrating shared themes for integrative analyses based on agreement between stakeholder specific coding trees and code books; which include non-identifying coded data and show the underlying quotes for each theme.

## Supplementary Information

Supplementary Information.

## Data Availability

Anonymised survey data (duplicates only removed) and focus group data (presented at theme level) is available online using the https://doi.org/10.17026/dans-xp4-j8t7.
