# Supplementary material for: Food system actor perspectives on future-proofing European food systems through plant breeding
Source: Sci Rep. 2023 Apr 3;13:5444. doi: 10.1038/s41598-023-32207-1 (PMC10069723; doi:10.1038/s41598-023-32207-1)
Supplement: Supplementary file 1 — Supplementary Information. [file 41598_2023_32207_MOESM1_ESM.docx]

# Supplementary 1

## Survey

### Translation and piloting

The survey was developed and piloted in English. A total of 17 participants piloted the English survey, with at least three testing each stakeholder specific survey variant. To access as many participants as feasible, it was translated and piloted in German and French using a modified TRAPD method ^2^. Six participants piloted the German and four the French versions, with at least one participant per language per survey variant. Changes made following pilot feedback included improved signposting, minor corrections to grammar and the updating of some terminology.

### Recruitment

Participants of the survey were volunteer stakeholders identified through the professional networks of the consortium belonging to each one of the predefined groups. Participants were asked to further distribute the survey link (snowball-sampling) to increase the number of participants. Project partners shared the survey links widely within their professional networks, on social media, and through direct contact with external organisations of relevance (such as the Food Climate Research Network, EAT forum, and IFPRI). The survey could be filled in by anyone who received the link. A total of 324 participants took part in the online survey (288 in English, 22 in French, and 14 in German). For information on the types of roles associated with each stakeholder group, see section one of the survey in Supplementary 6.

### Procedure

Participants were informed of the purpose and length of the survey, how the information they provided would be managed and their right to withdraw at the start of the survey. The investigators were named and professional email addresses listed in case of questions. The survey was open between April and May 2020. After informed consent the main survey (specific to the stakeholder group) started.

Surveys contained some specific question depending on the target stakeholder group which affected total survey length. Farmers, for example, were asked questions regarding their farm size and level of agricultural education to allow for comparisons with the target population. (See [Supplementary 6] for a copy of the survey in English for further detail regarding the precise questions included for each stakeholder stream.)

Some personal data was collected including: email addresses (collected so that participants could be kept up to date with the project), postal code (to determine the effects of socio-economic factors) and institution or company. IP addresses collected by *Qualtrics* were used to check for duplicates. To ensure the confidentiality of these data, several measures were in place. *Qualtrics* assures GDPR compliance and offers substantive data protection measures. Data were accessed and managed from encrypted, password-protected institutional cloud storage systems. All data submitted to [WUR repository] have been anonymised.

### Response and completion rates

A total of 324 participants took part in the online survey (288 in English, 22 in French, and 14 in German). Sixty-five responses were removed from analysis, as the respondents had not completed any data collection question blocks. Five survey responses were deleted as duplicate responses. This resulted in a total of 254 responses from participants who completed at least the first data collection segment of the survey (goal prioritisation). 201 respondents completed all core data collection segments (goal prioritisation and option card prioritisation): 39 for farm-level stakeholders, 26 for agri-business level stakeholders, 38 for consumer level stakeholders, and 98 for plant scientists. Of the 254 surveys, 120 had some missing data but were retained for analysis as the respondents had completed the initial data collection segment regarding goal prioritisation.  The majority of participants who responded regarding their location came from the UK (83), with additional participation from: Belgium (8), Croatia (1), Cyprus (2), Czech Republic (1), Denmark (2), France (15), Germany (11), Greece (1), Italy (31), Luxembourg (1), Netherlands (7), Portugal (2), Romania (1), Spain (10), and a further 12 responses from individuals currently living outside Europe.

## Focus groups

### Sampling frame and recruitment

The sampling frame was purposive. Participants were selected and approached on the basis of belonging to one of the pre-defined groups described above. Selection criteria were: 1) participants currently belong to one of the above professional groups, 2) Europe is the focus of participants’ work and 3) could consent to being involved in the online focus groups. Potential participants were recruited primarily via email, which included a poll to determine the most convenient date. Some farm-level participants were also contacted via a European agricultural association newsletter.

Forty five respondents indicated interest in participating in the focus groups. Interested participants were then sent a participant information sheet, which outlined the goals of the project, what the focus groups would involve and how personal data would be managed. Potential participants were asked to pass on the email invitation to other members of their organisation if they could not attend, as well as anyone they thought relevant to the project. A total of thirty five participants participated in one of ten focus groups (five with farm-level, two with agri-business, and three with consumer-level participants).

### Consent and data management

Prior to the start of the focus groups an online digital consent form was presented to participants which specified:

- Focus groups will be audio and video recorded
- Only the research team will have access to those recordings
- Contributions will be treated confidentially and participants will be pseudo-anonymised – any quotations used in reporting will be anonymised
- Data will be stored in a secure, password-protected location
- Participant’s right to withdraw from the study
- Any data belonging to the project would be destroyed after 10 years

### Hosting the focus groups

The focus groups were video and audio recorded via *Teams* with duplicate audio recordings made via Dictaphone as a back-up. Participants were made aware of the role of the moderator and the goals of the project via the participant information sheet and at the start of the focus groups. Ground rules were established that emphasised the importance of patience and turn-taking given the online format and lack of certain natural cues. Notes were recorded on a standardised form.

The focus groups lasted an average of 100 minutes, the longest being 125 and the shortest 70.

*Microsoft Teams* was used as a hosting platform alongside virtual whiteboarding website *MURAL* (www.mural.co). The 15 option cards and the empty Option Card #16 were incorporated into a whiteboard as a discussion tool, allowing “sticky notes” with suggestions to be added. Different copies of the whiteboard were made with randomised ordering of options to minimise anchoring bias. Focus group participants accessed the MURAL whiteboard via internet browser or smartphone without log in or account creation. Moderators shared their screens to guide participants through the option cards and to ensure recordings captured the visual elements of the discussion. More explicit cues had to be used to instigate group discussions as natural pauses or body language were suppressed by the online format.

Moderators for each stakeholder group had no existing relationships with any participants. In the agri-business focus groups, the project was introduced by a representative of *Euroseeds* (PJ), who has a professional relationship with several of the participants – after which the representative left before the actual focus group commenced. The moderators were experienced in the used interviewing technique (SS and JM in focus groups; AN in semi-structured interviewing).

### Data preparation

The video recordings of each focus group were sent to a private GDPR-compliant company for transcription – non-disclosure agreements were signed in advance. Once the transcripts had been returned, they were checked for errors and anonymised by removing identifying information.

# Supplementary 2

## Survey data: goal prioritisation

Percent of respondents choosing each goal as most, least or of in-between importance.

## Survey data: option prioritisation


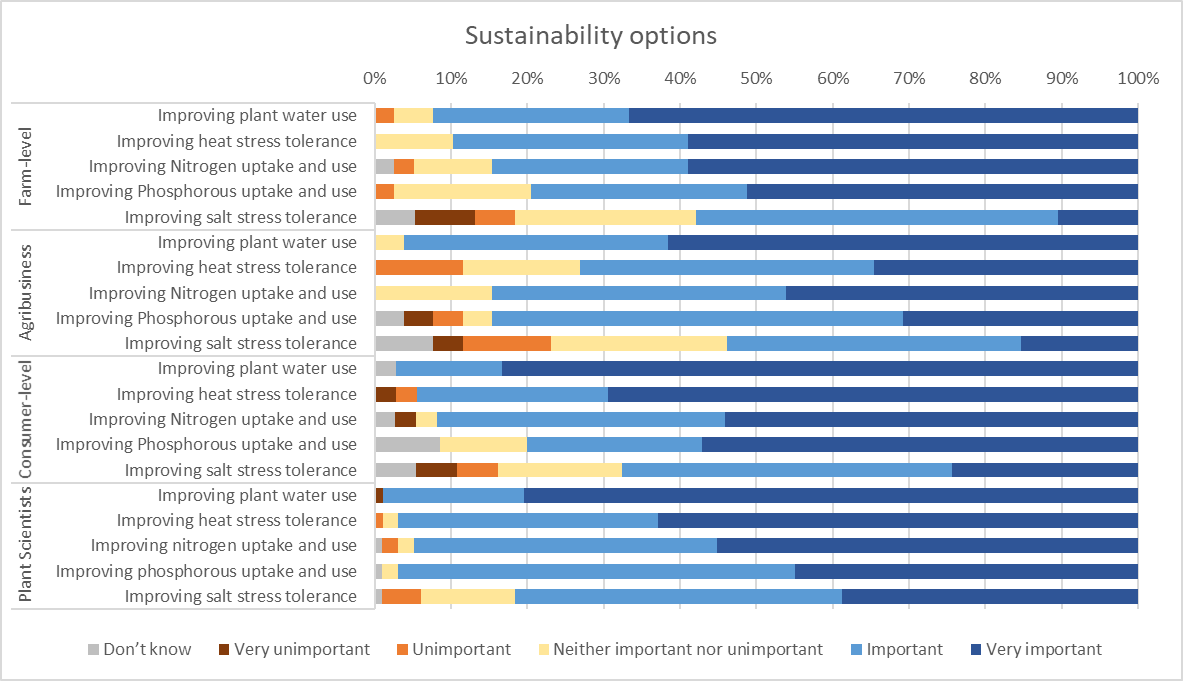


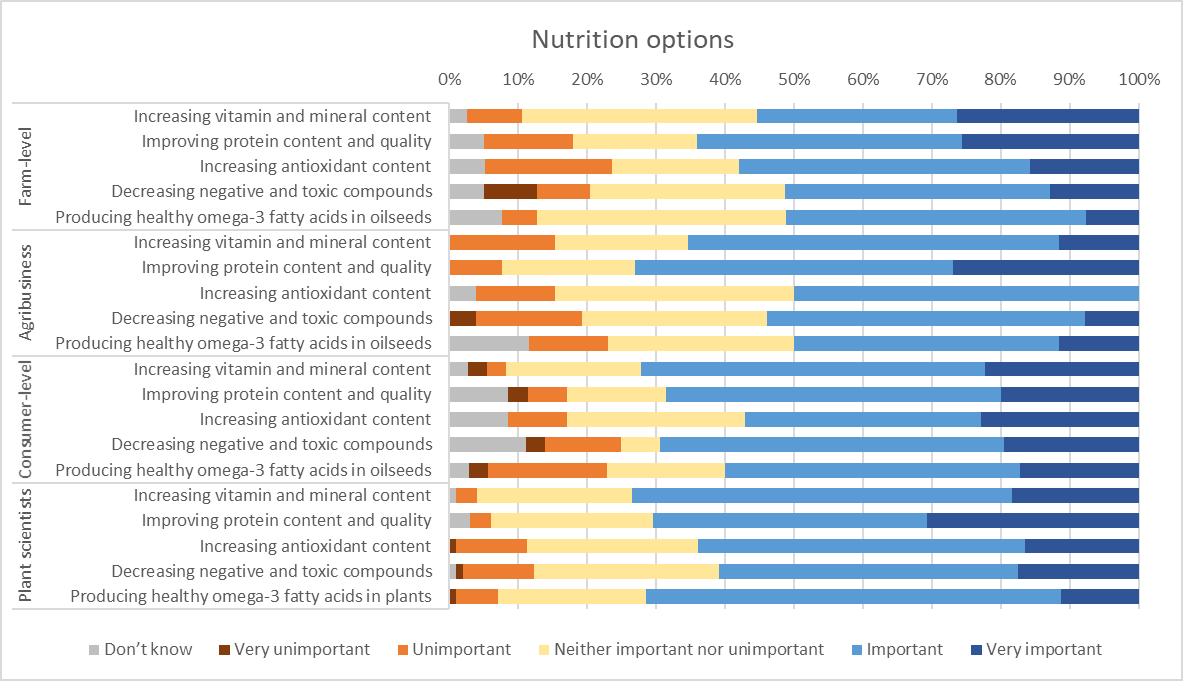


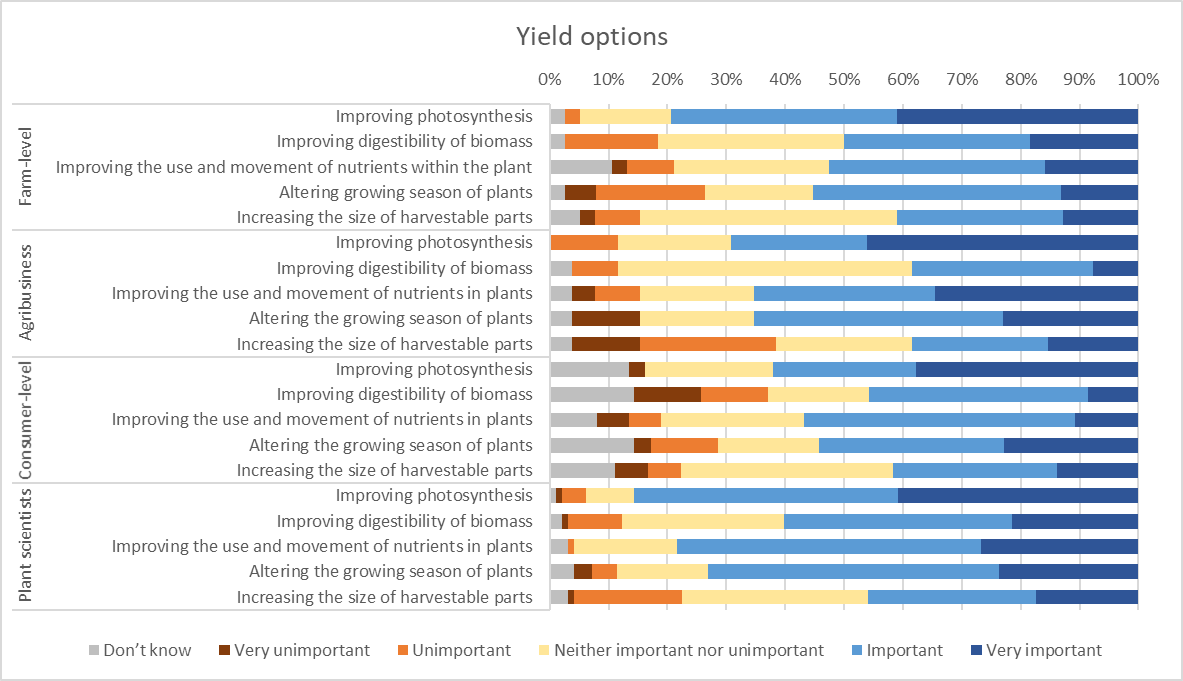


## Option card 16 data: surveys and focus groups

| Farm-level | |
| --- | --- |
| Survey | Focus groups |
| 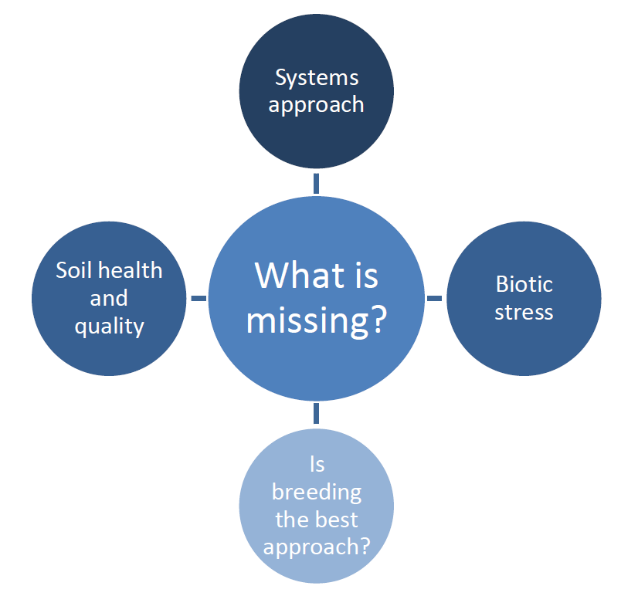 | 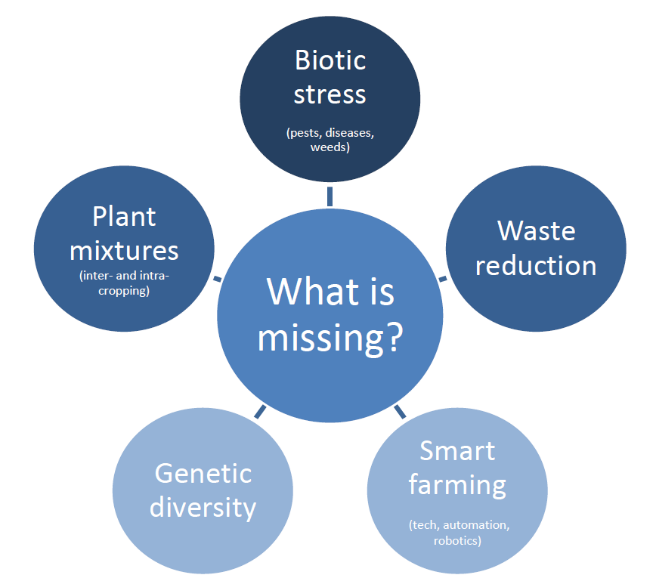 |

| Agribusiness | |
| --- | --- |
| Survey | Focus groups |
| 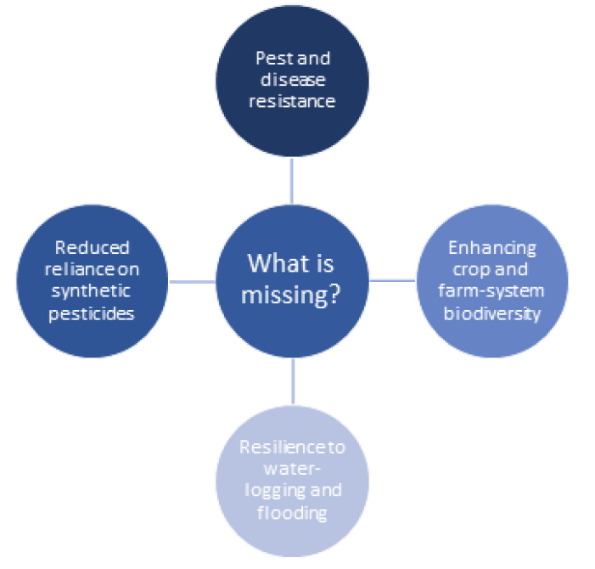 | 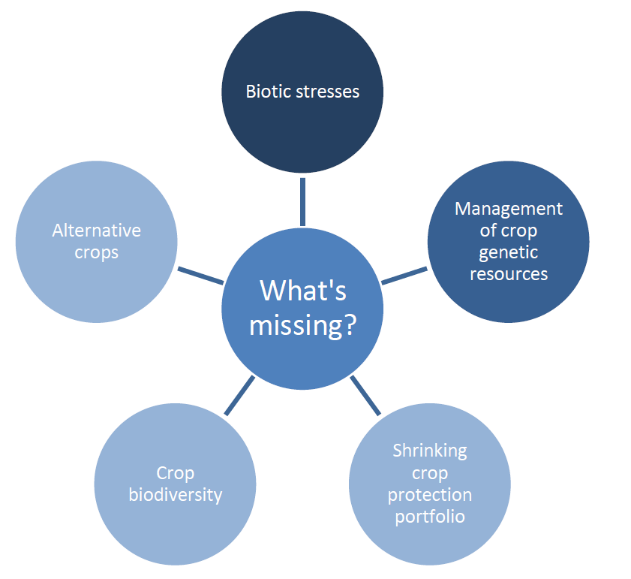 |

| Consumer-level | |
| --- | --- |
| Survey | Focus groups |
| 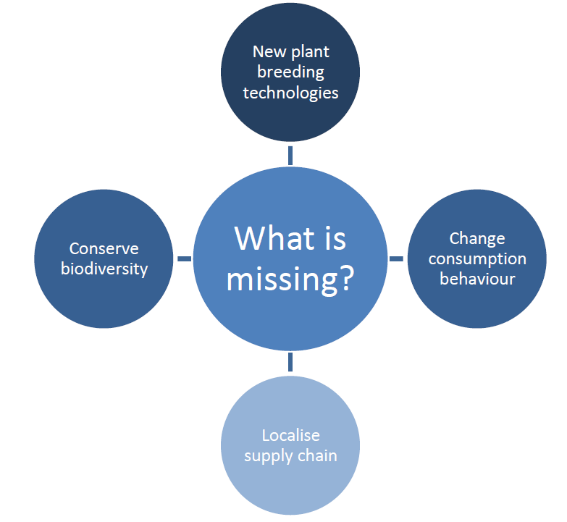 | 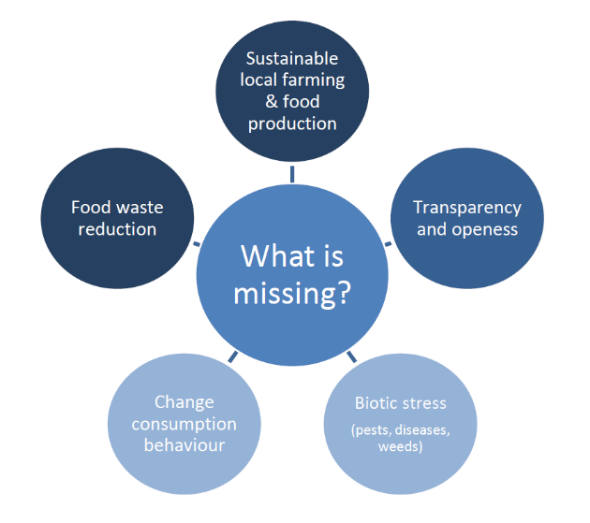 |

# Supplementary 3: Rapid evidence synthesis papers reviewed

# Supplementary 4: Emerging issues themes and example quotes

| **Theme** | **Farm-level** | **Agri-business** | **Consumer-level** |
| --- | --- | --- | --- |
| Alternatives | *“For me this one is easy. It is [heat] stress…It is one thing that I cannot manage. I think for me it is the most important because it is the one that I control the least” – Farmer representative #3* | *“I would ask, [does] it makes sense to increase carotenoid percentage in potato, instead of to grow carrots?” – Plant breeder #3* | *“… can also be algae and seaweed… as an alternative protein are really useful today.” – Consumer expert #1* |
| Tradeoffs | *“We all know that many of the genetic improvements or alterations we have done in crops, they came with a cost, like is it taste, or vitamins, or…more diseases or whatever? It always had a drawback on one side. – Policy stakeholder #1* | *“… my last comment would be on the last one, decreasing the negative and toxic compounds and elements. I would be extremely careful. I understand what the intent is, but we need to remember that these negatives are there for a reason, which is about the natural preservation [against] some kind of pest or insect...” – Agri-business expert #5* | *“I'm not excited about increasing the size of harvestable parts, and the reason is if increasing the size has negative impacts on quality or taste profile” – Consumer expert #5* |
| Resilience | *“So the probably upcoming effects of climate change and the desertification of many places in the European Union. That will be something important to consider, very, very important.” - Farmer representative #10* | *“As we mentioned, nitrogen uptake, phosphorus, so with the depleting global supplies for phosphorus are obviously important.” – Plant breeder #5* | *“I am also drawn to improving plant water use and heat stress tolerance because of the issue of global climate change, I can imagine that those would be ones that even most consumers would have a sense that this could be a problem if they thought about or if they think about climate change in any way.” - Consumer expert #5* |
| Interconnected breeding | *“I don’t like this idea of prioritising. I tell you straight out I’m very much against so-called trade-offs when it comes to breeding progress. There shouldn’t be. It is possible to have a plant that has improved nitrogen and phosphorous uptake.” - Farmer representative #9* | *“… if you want to improve photosynthesis, you need to be able to transport that additional fixed carbon, and then if there’s nowhere to load that additional energy or carbohydrates – for example if you don’t increase the size of the harvestable parts – then it would not accumulate either, or lead to yield. So there are several interconnected components of yield here.” – Plant breeder #5* | N/A |
| Variation and universality | *“There is one aspect I would like to stress, which is really important. We have to keep in mind that different regions have different needs and different characteristics. When we talk about sustainability we tend to use a general European concept that cannot be applied the same way in the northern, in the centre or in the southern parts.” - Farmer representative #1* | *“So I think [other participant] mentioned the fact about the market being important. So going back to the vitamin A story, this is absolutely a critical problem in Africa; in sub-Saharan Africa, and in Asia. But for the European environment, this is really a ‘nice to have’ for our populations.” – Plant breeder #5* | *“My first reaction is that it must be very different from regions within Europe, which traits that are most important.” – Consumer expert #2* |
| Value chain impacts | N/A | *“… if you had a high-antioxidant tomato or whatever, this would actually help to meet some of these dietary intakes, without changing dietary eating patterns.” – Plant breeder #5* | *“My thinking is more along the line of- when it comes to phosphorous and nitrogen, it will be like more related to the use of the efficient use of fertilizers or maybe also reducing the use of fertilizers, which might have like a big impact not only on the cost of production for farmers, as well as, the environmental implications of crop production.” - Consumer expert #1* |
| Markets | *“But the fact is that the market for the plant breeders, well it may look as if it is the growers, but in fact…[i]t is the processors, the buyers from the growers that determine.” – Farmer representative #1* | *“… you also have to consider what is the market you go for. Not only what could be done, what could also be sold.” – Plant breeder #6* | N/A |
| Food sovereignty | *“A lot of young people now and in 30 years’ time will probably be [vegan]... As long as we have a big import of soya for food and feed that is not a big problem I would say. But if you want to be self-sufficient on protein that is of course a big problem.” – Farmer representative #4* | *“… in relation to having in Europe more, paying more attention to producing locally protein crops… what you see is the gap in yield between crops like lupin, lentils or forage pea, against soy bean, that you can buy on the world market… we see how it works in Brazil; to increase the production, you just put down more trees, and you burn, and then you grow soy bean.” Plant breeder #2* | N/A |
| Knowledge exchange | *“I think the supply chain should be more involved in developing sustainable production systems. It should be a joint responsibility and you should not talk about resilient production systems. You should talk about resilient supply chains.” – Farmer representative #1* | N/A | *“… what you should be doing going out is telling the world these are the challenges you face, we face. What do you think about how we could be approaching what the pros and cons are and I think some way of making those issues more transparent to all parts of the community would help with being able to prioritize, because otherwise, I think that one's coming in with ignorance, because we can challenge and I think quite often we as a professional community don't offer means for people to engage in these things and then are sometimes surprised with people's ability to do so.” – Consumer expert #7* |
| Plant biotechnology and regulation | *“I think it is a good point also relating the whole question of accessibility of plant reproductive material for farmers. Now this is of course relevant with regards to patent protection. Also, plant variety rights maybe to a lesser extent, but also if we think about the farmer’s privilege to save and reuse seeds for own purpose.” - Farmer representative #2* | *“… it’s also a big problem that if, again, coming back to these new kind of breeding technologies, if Europe is allowed to import these foods or products made from these foods, then our farmers just don’t stand a chance, I think.” – Plant breeder #4* | *“The current legal framework in regulating GMO and others are still quite outdated in a way. So, if they really want to make a change in the food system and the agricultural system, I think they should consider revising the legal framework first ...” – Consumer expert #8* |
| Yield stability | *“In my perspective, the yield itself, it’s not really the major issue. The major issue, in my vision, it’s yield stability in the longer term. That’s what farmers look for, and that’s what the objective should be when we think to sustainable systems, in my opinion.” - Farmer representative #6* | *“In Germany and in many parts of northern Europe they are also already seeing the effects of heat stress and extended no rains in the regions during the summers.” – Agri-business expert #1* | N/A |
| Food system complexity | N/A | N/A | *“I would guess it's about knowing one's place in the world in terms of what range of the food supply they should be responsible for. And I think the recent events where we've had the pandemic have sort of highlighted some of those challenges. So, again, how to sort of feed into a complex system like this, and things around the governance and support that's available.” – Consumer expert #7* |
| Fostering innovation | N/A | N/A | *“…the regulation around both the technological innovations, but also a steering consumption patterns in a more sustainable direction I think are really difficult, but important aspects…” – Consumer expert #6* |
| Biotechnology acceptance | N/A | *“… when we talk about transparency towards the consumers and labelling, we only talk about being transparent on the technique and labelling the technique used. But if we are really truly transparent and we label as well what the improvements are that have been made thanks to the technique, what is the advantage that the consumer will receive thanks to this new product, then maybe the acceptance would be coming a little bit faster and with the full knowledge of what the product means.” – Agri-business expert #2* | *“…you think a range of breeding technologies… you think yes, that sounds really good. And when you dig deeper it's a genetic modification, there's going to be a lot of resistance from a consumer perspective to GM crops.” – Consumer expert #4* |
| Breeding for management | *It can support crop diversification. Crop diversification, it’s actually quite a good way to find a balance between biodiversity and the need of production, because, for example, by diversifying the diversity that is planted, so what are actually the crops, we can actually reach quite some good objectives in terms of agroecosystem services. - Farmer representative #6* | N/A | N/A |

# Supplementary 5: Examples of option cards and Option Card 16


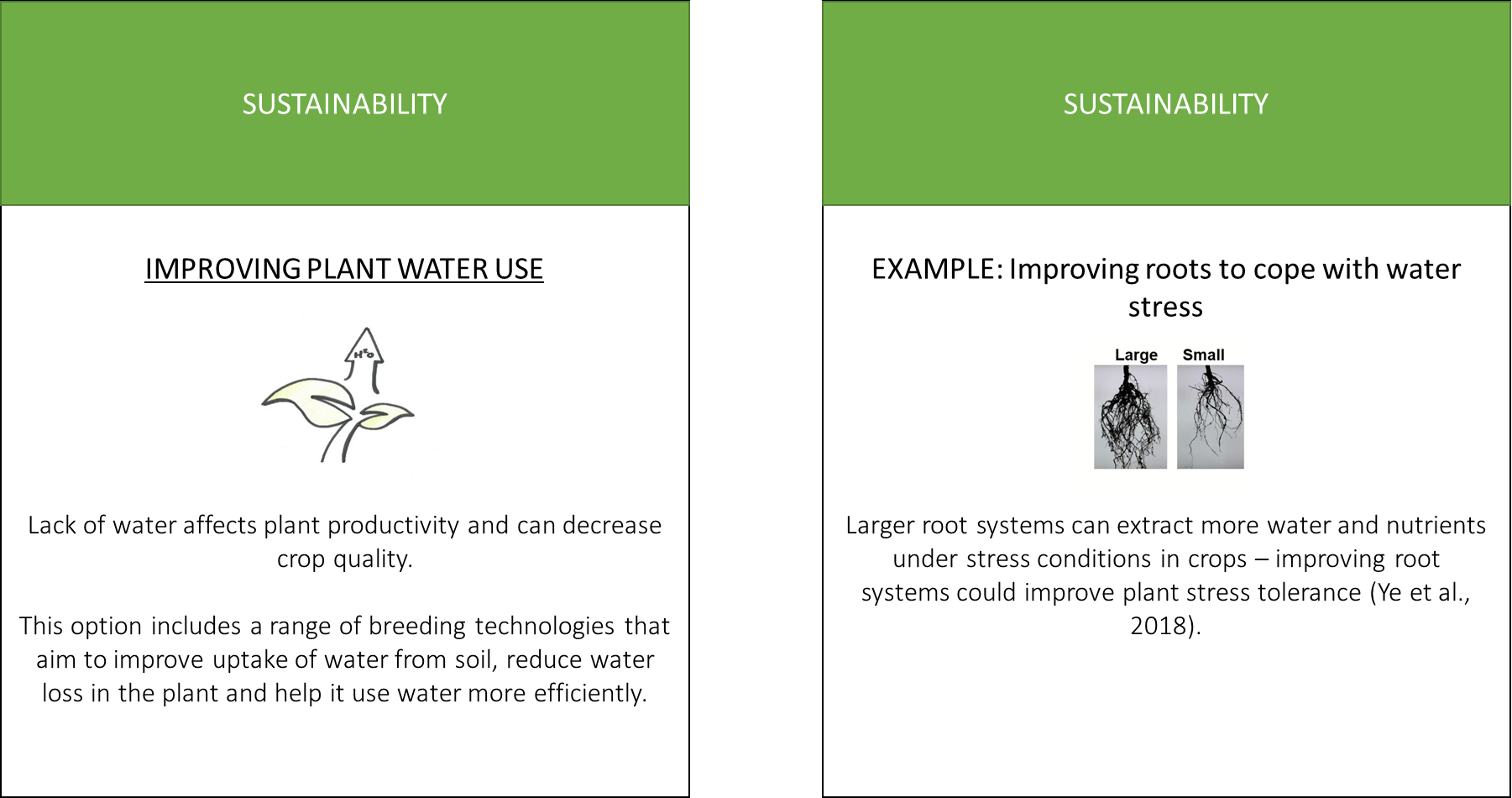


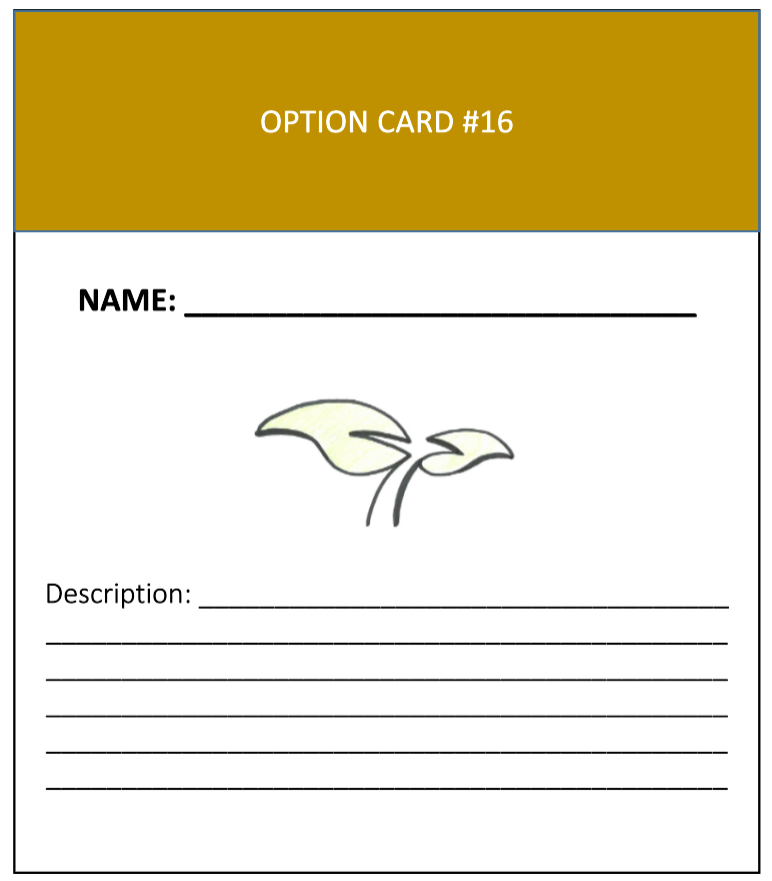


# Supplementary 6: Online survey

CropBooster-P Survey

Start of Block: Shared Demographic Questions

Q8 This survey is part of CropBooster-P, an EU project bringing together researchers and stakeholders across Europe to map and assess strategies for crop improvement.  You can find out more about the project on our website at [www.cropbooster-p.eu](https://www.cropbooster-p.eu).   As a member of the European food system, we want your opinions on the potential importance of several strategies for crop improvement that we have identified around improving the yield, nutritional quality, and sustainability of European crops.    By completing this survey you are agreeing to have your results analysed as part of this project.  Individual responses will be kept anonymous and will be used by the CropBooster-P team to better understand priorities for crop improvement in Europe.  They may also form the basis of publications.  Your data will be stored securely and anonymously and may be used in future research projects.  The results of this survey will be analysed by researchers at Lancaster University (United Kingdom) and Wageningen University (Netherlands).    You may request to have your response removed from the survey during the data collection phase. To do this, you must email iss@lancaster.ac.uk before 18 May 2020 with the email address you used when filling in the survey. Beyond this date, your data will no longer be able to be removed from the analysis.    If you have any questions, please do not hesitate to contact Dr Stacia Stetkiewicz, Dr Jonathan Menary, or Dr Abhishek Nair - s.stetkiewicz@lancaster.ac.uk; j.menary@lancaster.ac.uk; abhishek.nair@wur.nl.    [Click here to view the survey in French](https://lancasteruni.eu.qualtrics.com/jfe/form/SV_cOB8zuT8TOLgPWt) [Click here to view the survey in German](https://lancasteruni.eu.qualtrics.com/jfe/form/SV_4MgX5zsFynbrBm5)

Q3 What is your current age?

▼ Under 18 ... Over 100

Q9 Which of the following best describes you?

- Involved in farm-level activities
- Involved in agri-business or the food supply chain
- Consumer or consumer representative
- Plant scientist

End of Block: Shared Demographic Questions

Start of Block: Ranking

| 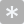 |
| --- |

Q38
In this section, you will be asked about how important different crop improvement options are in terms of future-proofing European crops.
 
Future-proofing crops is used to refer to improving crops in order to prepare them for the future needs of society and the challenges which will be faced by food systems between now and 2050.
  Please rank the following goals in terms of importance to future-proofing European crops, with 1 being most important and 3 least important.

______ Increasing yield

______ Improving nutritional quality

______ Improving sustainability

Q39 Please briefly describe why you have prioritised your chosen goal (in 1000 characters or less).

________________________________________________________________

________________________________________________________________

________________________________________________________________

________________________________________________________________

________________________________________________________________

Q42 Please indicate how important you feel each of the following options are for future-proofing European crops.

Q57
Please indicate how important you feel this option is for future-proofing European crops:

- Very unimportant
- Unimportant
- Neither important nor unimportant
- Important
- Very important
- Don't know

Q47
Please indicate how important you feel this option is for future-proofing European crops:
 

 

- Very unimportant
- Unimportant
- Neither important nor unimportant
- Important
- Very important
- Don't know

Q55
Please indicate how important you feel this option is for future-proofing European crops:

- Very unimportant
- Unimportant
- Neither important nor unimportant
- Important
- Very important
- Don't know

Q49
Please indicate how important you feel this option is for future-proofing European crops:

- Very unimportant
- Unimportant
- Neither important nor unimportant
- Important
- Very important
- Don't know

Q54
Please indicate how important you feel this option is for future-proofing European crops:

- Very unimportant
- Unimportant
- Neither important nor unimportant
- Important
- Very important
- Don't know

Q51
Please indicate how important you feel this option is for future-proofing European crops:

- Very unimportant
- Unimportant
- Neither important nor unimportant
- Important
- Very important
- Don't know

Q56
Please indicate how important you feel this option is for future-proofing European crops:

- Very unimportant
- Unimportant
- Neither important nor unimportant
- Important
- Very important
- Don't know

Q52
Please indicate how important you feel this option is for future-proofing European crops:

- Very unimportant
- Unimportant
- Neither important nor unimportant
- Important
- Very important
- Don't know

Q46
Please indicate how important you feel this option is for future-proofing European crops:

- Very unimportant
- Unimportant
- Neither important nor unimportant
- Important
- Very important
- Don't know

Q53
Please indicate how important you feel this option is for future-proofing European crops:

- Very unimportant
- Unimportant
- Neither important nor unimportant
- Important
- Very important
- Don't know

Q48
Please indicate how important you feel this option is for future-proofing European crops:
 

- Very unimportant
- Unimportant
- Neither important nor unimportant
- Important
- Very important
- Don't know

Q41
Please indicate how important you feel this option is for future-proofing European crops:

- Very unimportant
- Unimportant
- Neither important nor unimportant
- Important
- Very important
- Don't know

Q44
Please indicate how important you feel this option is for future-proofing European crops:

- Very unimportant
- Unimportant
- Neither important nor unimportant
- Important
- Very important
- Don't know

Q50
Please indicate how important you feel this option is for future-proofing European crops:

- Very unimportant
- Unimportant
- Neither important nor unimportant
- Important
- Very important
- Don't know

Q90
Please indicate how important you feel this option is for future-proofing European crops:
 

- Very unimportant
- Unimportant
- Neither important nor unimportant
- Important
- Very important
- Don't know

| 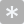 |
| --- |

Q59 Are there any other goals which were not included in the above list, but which you feel are important for future-proofing crops? If so, please provide a brief description below. (in 1000 characters or less)

________________________________________________________________

________________________________________________________________

________________________________________________________________

________________________________________________________________

________________________________________________________________

End of Block: Ranking

Start of Block: Shared demographic questions part 2

Q2 Are you contributing to a CropBooster-P focus group in spring 2020?

- Yes
- No
- Unsure
- Other, please specify: ________________________________________________

| Page Break |  |
| --- | --- |

Q1 Capacity in which you are filling in this survey (this could be your job title, an organisation you represent, or simply as an interested individual)

________________________________________________________________

________________________________________________________________

________________________________________________________________

________________________________________________________________

________________________________________________________________

| Page Break |  |
| --- | --- |

Q4 What is your sex?

 Why are we asking? - We are collecting this information in order to check the representation of different age, gender, and geographic groups in our survey - for example, if the majority of our responses are coming from one particular region of Europe, this might be important when interpreting our results.

- Male
- Female
- Prefer not to say

Q5 What is your home postcode? (UK respondents, please give at least the first three characters of your postcode)

________________________________________________________________

| Page Break |  |
| --- | --- |

Q6 Formal education level (please indicate the highest applicable to you - hover over the answer choices for examples / or [click here](https://lancasteruni.eu.qualtrics.com/CP/File.php?F=F_0UpqXAgd0IrOsT3) for examples)

- Less than primary education
- Primary education
- Lower secondary education
- Upper secondary education
- Post-secondary non-tertiary education
- Short-cycle tertiary education
- Bachelor’s or equivalent degree
- Master’s or equivalent degree
- Doctoral or equivalent degree
- Other, please specify: ________________________________________________

| Page Break |  |
| --- | --- |

Q78 Which country do you live in? (if you split your time between multiple countries, please indicate the country of your primary residence)

- Austria
- Belgium
- Bulgaria
- Croatia
- Cyprus
- Czech Republic
- Denmark
- Estonia
- Finland
- France
- Germany
- Greece
- Hungary
- Iceland
- Ireland
- Italy
- Latvia
- Lithuania
- Luxembourg
- Malta
- Montenegro
- Netherlands
- Norway
- Poland
- Portugal
- Romania
- Slovakia
- Slovenia
- Spain
- Sweden
- Switzerland
- United Kingdom
- Other, please specify: ________________________________________________

| Page Break |  |
| --- | --- |

| 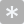 |
| --- |

Q15 Which crops do you feel are most important for the future of European agriculture?  (choose up to 5)

- Barley
- Carrots
- Grain maize and corn-cob mix
- Grapes
- Oats
- Olives
- Onions
- Potatoes
- Rape and turnip rape seeds
- Rice
- Rye and maslin
- Sorghum
- Soya
- Spelt
- Sugar beet
- Sunflower seeds
- Tomatoes
- Triticale
- Wheat
- Other, please specify: ________________________________________________

End of Block: Shared demographic questions part 2

Start of Block: Farm level questions

Q11 Which of the following best describes you:

- Farmer
- Farmer representative
- Farm support/advisor
- Environmental regulator or policy maker
- Scientific expert in resource use efficiency, environmental impacts, etc.
- NGO with a focus on farm-level concerns, such as the environmental impacts of farming
- Other farm-level stakeholder, please specify: ________________________________________________

| Page Break |  |
| --- | --- |

Q12 What is your highest level of agricultural education?

- Only practical experience on-farm
- Basic agricultural training (this includes a completed agricultural apprenticeship)
- Full agricultural training (two or more years of full-time higher education)
- Other, please specify: ________________________________________________
- Not applicable

| Page Break |  |
| --- | --- |

Q13 Is your farm mixed animal and crop farming, or solely crops?

- Mixed crop and animal farming
- Crop specialist
- Animal specialist
- Not applicable

| Page Break |  |
| --- | --- |

Q14 What types of farms do you primarily represent/work with?

- Mixed crop and animal farming
- Crop specialists
- Animal specialists
- Not applicable

| Page Break |  |
| --- | --- |

| 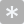 |
| --- |

Q16 Which crops do you primarily work with or on?  (choose up to five)

- Barley
- Carrots
- Grain maize and corn-cob mix
- Grapes
- Oats
- Olives
- Onions
- Potatoes
- Rape and turnip rape seeds
- Rice
- Rye and maslin
- Sorghum
- Soya
- Spelt
- Sugar beet
- Sunflower seeds
- Tomatoes
- Triticale
- Wheat
- Other, please specify: ________________________________________________
- Not applicable

| Page Break |  |
| --- | --- |

Q17 What country is your farm located in?

- Austria
- Belgium
- Bulgaria
- Croatia
- Cyprus
- Czech Republic
- Denmark
- Estonia
- Finland
- France
- Germany
- Greece
- Hungary
- Iceland
- Ireland
- Italy
- Latvia
- Lithuania
- Luxembourg
- Malta
- Montenegro
- Netherlands
- Norway
- Poland
- Portugal
- Romania
- Slovakia
- Slovenia
- Spain
- Sweden
- Switzerland
- United Kingdom
- Other, please specify: ________________________________________________

| Page Break |  |
| --- | --- |

Q18 What size is your farm in total? (including rented land)

- 0 – less than 2 ha
- 2 – 4.9 ha
- 5 – 9.9 ha
- 10 – 19.9 ha
- 20 – 29.9 ha
- 30 – 49.9 ha
- 50 – 99.9 ha
- 100 ha or over

| Page Break |  |
| --- | --- |

| 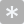 |
| --- |

Q19 What are the current primary markets for your crops?  (choose up to three)

- Animal Feed
- Human food
- Fuel
- Drinks industry
- Other, please specify: ________________________________________________

| Page Break |  |
| --- | --- |

Q20 Does your farm have any specific certifications or organisational affiliations, or are you a member of any specific agri-environmental schemes, such as Organic, LEAF, etc? (please indicate any which apply, even if they do not apply to your entire farm)

- Yes, please specify: ________________________________________________
- No
- Unsure

| Page Break |  |
| --- | --- |

Q21 Do you own or rent your farm?

- Own
- Rent
- Own some, rent some (please specify approximate hectares for each) ________________________________________________
- Other, please specify ________________________________________________

| Page Break |  |
| --- | --- |

Q22 Which country do you primarily work in?

- Austria
- Belgium
- Bulgaria
- Croatia
- Cyprus
- Czech Republic
- Denmark
- Estonia
- Finland
- France
- Germany
- Greece
- Hungary
- Iceland
- Ireland
- Italy
- Latvia
- Lithuania
- Luxembourg
- Malta
- Montenegro
- Netherlands
- Norway
- Poland
- Portugal
- Romania
- Slovakia
- Slovenia
- Spain
- Sweden
- Switzerland
- United Kingdom
- Other, please specify: ________________________________________________

| Page Break |  |
| --- | --- |

Q23 If you are a farm advisor, or frequently work with farmers, what is the average size of farm you usually work with?

- 0 – less than 2 ha
- 2 – 4.9 ha
- 5 – 9.9 ha
- 10 – 19.9 ha
- 20 – 29.9 ha
- 30 – 49.9 ha
- 50 – 99.9 ha
- 100 ha or over
- Unsure
- Not applicable

| Page Break |  |
| --- | --- |

| 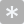 |
| --- |

Q24 What are the primary markets for the crops you usually work with or on?  (choose up to three)

- Animal Feed
- Human food
- Fuel
- Drinks industry
- Other, please specify: ________________________________________________
- Unsure
- Not applicable

| Page Break |  |
| --- | --- |

Q25 Which of the following best describes the company or organisation you work for/are a part of?

- Farm/farmer
- NGO
- Research institute
- Higher education institute
- Business
- Farm advisory group
- Other, please specify: ________________________________________________

End of Block: Farm level questions

Start of Block: Business level questions

Q26 Which of the following best describes you?

- Agri-food business member (other than farmers)
- Agri-food business representative
- Trade or supply chain expert
- Agricultural technology expert
- Agricultural economist
- Plant breeder
- NGO with a focus on business-level concerns, such as sharing of genetic material for breeding
- Other business-level stakeholder, please specify: ________________________________________________

| Page Break |  |
| --- | --- |

| 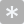 |
| --- |

Q28 Which crops do you primarily work with or on?  (choose up to 5)

- Barley
- Carrots
- Grain maize and corn-cob mix
- Grapes
- Oats
- Olives
- Onions
- Potatoes
- Rape and turnip rape seeds
- Rice
- Rye and maslin
- Sorghum
- Soya
- Spelt
- Sugar beet
- Sunflower seeds
- Tomatoes
- Triticale
- Wheat
- Other, please specify: ________________________________________________
- Not applicable

| Page Break |  |
| --- | --- |

Q29 Which country do you primarily work in?

- Austria
- Belgium
- Bulgaria
- Croatia
- Cyprus
- Czech Republic
- Denmark
- Estonia
- Finland
- France
- Germany
- Greece
- Hungary
- Iceland
- Ireland
- Italy
- Latvia
- Lithuania
- Luxembourg
- Malta
- Montenegro
- Netherlands
- Norway
- Poland
- Portugal
- Romania
- Slovakia
- Slovenia
- Spain
- Sweden
- Switzerland
- United Kingdom
- Other, please specify: ________________________________________________

| Page Break |  |
| --- | --- |

Q30 Which of the following best describes the company or organisation you work for:

- Seed breeding and supply
- Fertiliser or chemical input supplier
- Non-governmental organisation or advocacy
- Processing or packaging
- Food safety
- Agricultural economics research institute
- Retail or distribution
- Other, please specify: ________________________________________________

End of Block: Business level questions

Start of Block: Consumer level questions

Q31 Which of the following best describes you?

- Consumer body representative
- Consumer research agency representatives
- Expert in consumer behaviour and choice
- Individual
- NGO with a focus on consumer-level concerns, such as consumer awareness campaigns
- Other, please specify: ________________________________________________

| Page Break |  |
| --- | --- |

Q32 Which country do you primarily work in?

- Austria
- Belgium
- Bulgaria
- Croatia
- Cyprus
- Czech Republic
- Denmark
- Estonia
- Finland
- France
- Germany
- Greece
- Hungary
- Iceland
- Ireland
- Italy
- Latvia
- Lithuania
- Luxembourg
- Malta
- Montenegro
- Netherlands
- Norway
- Poland
- Portugal
- Romania
- Slovakia
- Slovenia
- Spain
- Sweden
- Switzerland
- United Kingdom
- Other, please specify: ________________________________________________

| Page Break |  |
| --- | --- |

Q33 Which of the following best describes the company or organisation you work for?

- NGO
- Consumer representation group
- Consumer research agency
- Research institute investigating consumer behaviour and choice
- Other, please specify: ________________________________________________
- Not applicable: I am an individual, completing this survey in my capacity as a consumer

End of Block: Consumer level questions

Start of Block: Plant Scientist questions

Q40 Which of the following best describes the company or organisation you work for?

- University
- Public research institute
- Private research institute
- NGO
- Other, please specify: ________________________________________________

| Page Break |  |
| --- | --- |

| 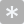 |
| --- |

Q34 Which crops do you primarily work with/on? (choose up to 5)

- Barley
- Carrots
- Grain maize and corn-cob mix
- Grapes
- Oats
- Olives
- Onions
- Potatoes
- Rape and turnip rape seeds
- Rice
- Rye and maslin
- Sorghum
- Soya
- Spelt
- Sugar beet
- Sunflower seeds
- Tomatoes
- Triticale
- Wheat
- Other, please specify: ________________________________________________
- Not applicable

| Page Break |  |
| --- | --- |

Q35 Which country do you primarily work in?

- Austria
- Belgium
- Bulgaria
- Croatia
- Cyprus
- Czech Republic
- Denmark
- Estonia
- Finland
- France
- Germany
- Greece
- Hungary
- Iceland
- Ireland
- Italy
- Latvia
- Lithuania
- Luxembourg
- Malta
- Montenegro
- Netherlands
- Norway
- Poland
- Portugal
- Romania
- Slovakia
- Slovenia
- Spain
- Sweden
- Switzerland
- United Kingdom
- Other, please specify: ________________________________________________

| Page Break |  |
| --- | --- |

Q77 Are you directly involved with the CropBooster-P project?

- Yes
- No
- Other, please specify: ________________________________________________

End of Block: Plant Scientist questions

Start of Block: Thank you

| 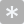 |
| --- |

Q74 Any other comments?

________________________________________________________________

________________________________________________________________

________________________________________________________________

________________________________________________________________

________________________________________________________________

| 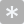 |
| --- |

Q76 If you would like to receive information about the results of this project directly, please leave your email address below.   Your input will always remain anonymous.

________________________________________________________________

Q75 Thank you for taking the time to complete this survey.     **Please share this survey!**   We are looking for as many responses and views on these issues as possible, so that we can provide useful data to the EU about priorities for future research in crop breeding.  Please consider sharing this survey with colleagues, friends, and connections anywhere in Europe – [a sharing link to the survey is available here](https://lancasteruni.eu.qualtrics.com/jfe/form/SV_8qcXjX7Y7gMkN49), and a QR code is below.  The survey is available in [English](https://lancasteruni.eu.qualtrics.com/jfe/form/SV_8qcXjX7Y7gMkN49), [French](https://lancasteruni.eu.qualtrics.com/jfe/form/SV_cOB8zuT8TOLgPWt), and [German](https://lancasteruni.eu.qualtrics.com/jfe/form/SV_4MgX5zsFynbrBm5).  Thank you for your support.    QR code link to the survey

End of Block: Thank you

# Supplementary 7: Focus group protocol

|  | **Online workshop outline** | The purpose of these workshops is to understand the potential economic, social and environmental impacts of CropBooster-P crop improvement options, which fall under three headings: yield, nutrition and sustainability.    The workshops are an opportunity for stakeholders – farmers, NGOs, breeders, agri-food industry and others – to discuss issues around these options and feed into a roadmap for the future.    We will be showing stakeholders 15 crop improvement options identified by CropBooster scientists as possible and desirable for future plant breeding efforts. These are:  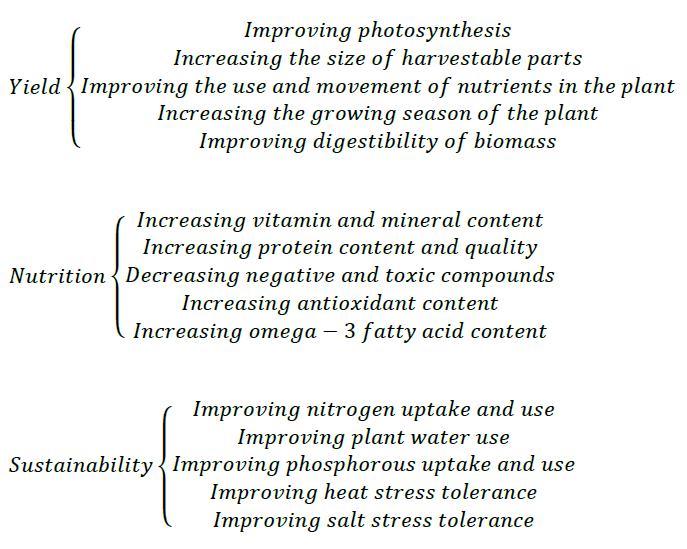    The workshops will also help us determine which options will be explored in a systematic literature review later. |
| --- | --- | --- |
|  | **Main questions** | 1. *What are the CropBooster option priorities for key stakeholder groups?* 2. *What are the potential social, economic and environmental impacts of the CropBooster options?* 3. *What important issues do the CropBooster options leave out?* |
|  | **Details** | There will be up to 15 online workshops:     1. *2.1A: farmers & farmer representatives (X3)* 2. *2.1B: regulators, policy makers and NGOs (X3)* 3. *2.2A: plant breeders (X3)* 4. *2.2B: agri-food supply chain (X3)* 5. *2.3A: consumer interest organisations (X3)*     We are aiming for 4-5 participants at each online workshop, which will be moderated by one of three postdoctoral researchers in charge of recruiting for and hosting the event    Participants will cycle through three “virtual stations” on *Mural* before moving onto a final activity, Option Card #16. Moderators must begin the workshops at a different station every time.    The stations will represent either yield, nutrition or sustainability. At each station there will be four or five ‘option cards’ (see below) that describe one of the options for that station:    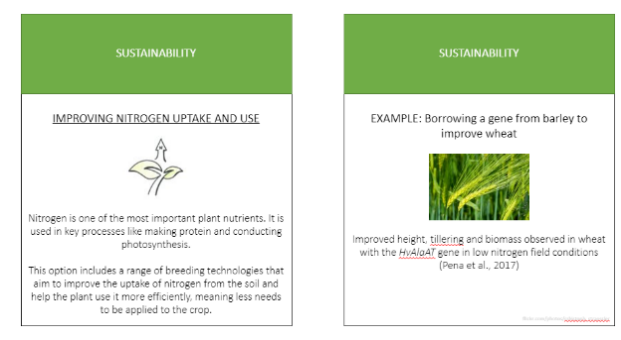    To facilitate this process, six *Mural* whiteboards have been created, each with different station and option card ordering. This has been done to reduce any *order bias* and the effects of tiredness as participants move through the session. |
|  | **Materials** | Make sure:     - You have sent the PIS to all participants by email at least 24 hours in advance of the online workshop; preferable attached to the invitation email. - You have created the event as a Teams meeting (this is mandatory for video recording) - You have created a back-up meeting in Webex - You have a draft of an email to all participants with the back up Webex link prepared and ready to be sent in case of any issues with Teams - You have sent a follow-up email that details the **time**, Teams **link** and **agenda** for the meeting - Make sure: - You have **screen capture software** set up or a **voice recorder to record audio via laptop/tablet speakers (this is back up in case Teams doesn’t record properly)** - You know how to use the voice recorder - You have checked that the voice recorders work (**battery**) - You have provided participants with a link to consent form - You have checked in advance that all participants have filled in the online consent form - Have links to consent forms ready in case anyone has not yet done it/wants to remind themselves of what was in it - You have links to option card materials and are comfortable using them - You have a **note pad** - **You have the printed/written out notetaking sheet** - **You have two pens** - List of (expected) attendees - Partner organisation is either A. attending to give a short presentation, B. sending a prepared video which you have ready, or C. not attending and you have added a thank you slide to the presentation - You have a spare computer already switched on, with the links for the Teams and Webex calls ready to activate if need be - You have an LAN to connect to the internet directly - You have a set of headphones (preferably with a microphone) - unless you are using the dictaphone as a back up, in which case check that your audio quality is acceptable - You know who is attending and who is missing |
|  |  |  |
| Allow ~30 minutes for people to arrive and mingle | **Before starting** | Ensure that you:     - Greet people as they arrive and make them feel welcome - Chat with them, try not to leave anyone out - You explain to participants that you will be recording the event - Check everyone’s microphone and video connections individually - We have a designated backup moderator ready to help out - Send out a link to consent forms in advance of the meeting |
| 1-15 | **Welcome presentation** | - Hosting partner can give a quick introduction (1-2 minutes) or provide a video - Explain project - Focussing on three areas of crop improvement: yield, nutrition and sustainability - Explain ground rules - There are no wrong answers - We’re video/audio recording so we don’t miss anything but your responses will be kept anonymous - Online meetings aren’t as fluid as in-person meetings, so please be patient with each other and I’ll try to make sure everyone gets a turn speaking. - Glitches usually resolve quickly – here's how we will deal with them - If you have issues with audio during the call, please use the chat function to alert the moderator - If the moderator drops out of the call and does not return within 5 minutes, please: 1) check your email to see if we have sent you anything and if not, 2) contact the emergency moderator (put the emergency moderator’s email in the chat)     **[REMIND EVERYONE THAT THEY NEED TO SIGN THE CONSENT FORM IF THEY HAVEN’T DONE SO]** |
| 15-20 | **Introduction (only at option station #1)** | **JM: YIELD STATION**  **AN: NUTRITION**  **SS: SUSTAINABILITY**    **[START TEAMS RECORDING AND VOICE RECORDER/SCREEN CAPTURE SOFTWARE]**    I would like each person to briefly introduce themselves:     1. Can you tell us your first name and a little about your organisation?     **[MAKE A NOTE OF PEOPLE’S NAMES – YOU’LL NEED THEM]** |
| 20-25 | **Warm-up question** | OK, now I would like to ask about what you think about the challenges for European food and agriculture:     1. What do you think the biggest challenges will be over the next 30 years? |
| 25-45 | **Appraisal of Cropbooster options and impact assessment** | **[PROVIDE A LINK (ABOVE) TO THE APPROPRIATE MURAL START – EXPLAIN THAT YOU WILL ALSO SHARE YOUR SCREEN. ENSURE EVERYONE CAN SEE OPTION CARDS]**    Here are some targets for crop improvement that our team have highlighted as important. We’ll go over them together but it might be useful to make a note of those you find interesting.    **[ALLOW EVERYONE TO READ THE CARDS]**    **[ON ‘SUMMARY PAGE’ ASK PARTICIPANTS TO MAKE A NOTE OF WHICH OPTION THEY THINK IS MOST AND WHICH LEAST IMPORTANT]**     1. Which option strikes you as the most important? Which option is least important?     **PROMPT:**    **WHY IS [OPTION] THE MOST IMPORTANT/UNIMPORTANT?**    **DID ANYONE ELSE HAVE THAT OPTION AS THE MOST/LEAST IMPORTANT?**    **NOBODY HAS SAID [OPTION]. WHY?**    Now, thinking about the potential impacts of these options:     1. What would be the impact of [option] be?     **PROBE:**    **WHY DO YOU THINK THAT?**    **WHAT ABOUT [SOCIAL/ECOMOMIC/ENVIRONMENTAL] IMPACTS?**    **PROMPT:**    **DOES ANYONE DISAGREE ABOUT THE IMPACT OF THAT OPTION**    **WHAT ELSE WOULD HAVE TO HAPPEN FOR [OPTION] TO HAVE IMPACT?** |
| 45-50 | **Insurance question** | Lastly, I would like to know:     1. How do these options meet the challenges you outlined earlier? |
| 50-80 | **Option station #2** | **[MOVE GROUP TO NEXT OPTION CATEGORY]** |
| 80-110 | **Option station #3** | **[MOVE GROUP TO NEXT OPTION CATEGORY]** |
| 110-125 | **Option Card #16 activity** | Now you have an opportunity to tell us what **else** should be included in these options for future-proofing European agriculture.    **[SCROLL TO OPTION CARD #16 AND ASK THEM TO DISCUSS WHAT SHOULD BE ON IT]**    **PROMPT:**    **CAN WE AGREE ON WHAT OPTION #16 SHOULD INCLUDE?**    **WHAT PROBLEM WAS RUNNING THROUGH PREVIOUS DISCUSSIONS?**    **[YOU CAN ADD A POST-IT NOTE BY DOUBLE-CLICKING IN MURAL]** |
|  | **Debrief** | - Inform participants that you have now reached the end of the formal workshop. - Ask if they have any remaining questions. - Thank participants for their time and tell them ways in which they can stay in touch. - Mention the integrative workshop and/or second workshop.     **[END RECORDING]** |
|  | **Contingencies** | 1. What should I do if a participant(s) do not join the online workshop? What is the minimum number which we will run the call with?     At <2 participants, switch to an alternative protocol.     1. What should I do if Teams does not work?     Send participants a link to Webex (or other backup software).     1. What should I do if neither Teams nor the back up software works?     Ask backup moderator if they can take over or find another suitable date with participants by email.     1. What should I do if there is a glitch and a participant drops out?     Continue and make a note of when they left the call – if they manage to reconnect, then bring them up to speed with what has been said. Invite them to join a subsequent workshop (if possible).     1. What should I do if there is a glitch and the moderator drops out temporarily?     Send them a chat/email informing participants that you will reconnect.  If you cannot reconnect after 5 minutes, inform the back-up moderator and ask them to take over.     1. What should I do if a participant’s video does not work?     Continue with audio only.     1. What should I do if a participant’s audio does not work?     Ask them to reconnect – if problem persists, ask them to check their audio settings. Invite them (by chat/email) to subsequent workshop.     1. What should I do if one or more participants can’t use Mural/see the option cards?     Use screensharing – if fidelity is still too low, send the option card PPT slides to the Teams group.     1. What should I do if a voice recorder does not work?     Use your mobile phone to record audio (most have applications for dedicated audio recording, otherwise record a video).     1. What should I do if too many participants come to the event?     Take their details, give them a name tag and have them join any of the other focus groups.     1. What should I do if someone is very late?       If they join before or while the group is reviewing the options for the first category, allow them to join and bring them up to speed while the rest of the group reviews the options, giving them time to look at these as well.  If they join after this point, ask them to join another focus group at a later date.     1. What should I do if there is a fire alarm or other emergency during the call?     Inform participants that this is not a drill and tell them that you will have to leave the building and that the back up moderator will take over shortly. Ask them to wait in the call and review the option cards for that section while they wait.  Exit the building, bringing the voice recorder and laptop with you. Once safe, contact the back up moderator and ask them to take over the call if your participants have not already done so.     1. What should I do if one person is dominating the focus group?     Start by asking for direct responses from other participants (e.g. “Does anyone have a different view?”). If it persists, you can directly ask the disruptive person to give others a chance to speak or throw them a stern look. As a last resort, they can be asked to leave. |
|  | **Transcription** | Video/audio files should be uploaded to the secure shared drive ASAP in the following format:    *[moderator initials] – [date] – [workshop #] – [number participants]*    Example: **JM – 09032020 – 22A - 4** |
